# Comprehensive Approaches to Urolithiasis in Renal Transplants: A Narrative Review

**DOI:** 10.3390/jcm13144268

**Published:** 2024-07-22

**Authors:** Catalina Solano, Mariela Corrales, Frédéric Panthier, Steeve Doizi, Olivier Traxer

**Affiliations:** 1 GRC n20, Groupe de Recherche Clinique sur la Lithiase Urinaire, Tenon Hospital, Sorbonne University, 75020 Paris, France; mariela_corrales_a@hotmail.com (M.C.); frederic.panthier@aphp.fr (F.P.); steeve.doizi@gmail.com (S.D.); olivier.traxer@aphp.fr (O.T.); 2 Uroclin S.A.S., Department of Endourology, Medellín 050021, Colombia

**Keywords:** kidney transplantation, ureteroscopy, percutaneous nephrolithotomy, urolithiasis

## Abstract

This review addresses the management of urolithiasis in renal transplant recipients, a notably vulnerable group due to the unique anatomical and physiological alterations of the transplanted organ. The prevalence of nephrolithiasis in these patients varies between 0.1% and 6.3%, with a significant impact on graft longevity and function. Surgical access complications due to the renal graft’s position on the iliac vessels and the variety of urinary anastomoses complicate the treatment approaches. This study evaluates the effectiveness and outcomes of percutaneous nephrolithotomy (PCNL) and ureteroscopy (URS), two primary minimally invasive strategies for managing graft stones. Through a narrative review using the PubMed and EMBASE databases, it was found that PCNL offers high stone-free rates especially beneficial for large stones, whereas URS provides a less invasive option with a lower risk of complications for small stones. Both techniques require tailored approaches based on stone composition—mostly calcium oxalate—and specific patient anatomical factors. This review underscores the importance of early diagnosis, appropriate treatment selection, and continuous post-treatment monitoring to mitigate risks and promote long-term renal function in transplant recipients.

## 1. Introduction

Urolithiasis in renal transplant recipients represents a unique clinical challenge due to the altered anatomical and physiological conditions of the transplanted organ. With an incidence rate varying from 0.1% to 6.3% [1,2,3,4], kidney stones in transplanted kidneys not only are rare but also pose significant risks to the longevity and functionality of the graft. The heterotopic position of the renal graft on the iliac vessels complicates the surgical access for stone removal, and diverse urinary anastomosis techniques such as those for ureterovesical and pyeloureteral anastomosis complicate the clinical approach. Early recognition and management of nephrolithiasis post-transplant are vital for maintaining graft function and patient health.

The management of kidney stones in the transplanted kidney is multifaceted, involving a range of minimally invasive techniques tailored to the specifics of the transplant anatomy. Treatment options include extracorporeal shock wave lithotripsy (ESWL), URS, and percutaneous or combined surgical approaches. Each technique has its characteristics regarding invasiveness, risk of complications, and outcomes. Factors such as stone composition—predominantly calcium oxalate—and location within the graft significantly influence the choice of treatment [5]. The overarching goal is to achieve stone clearance with minimal compromise to the kidney’s integrity and function.

Despite the complexities involved, the current strategies for managing urolithiasis in transplanted kidneys show promising results, with most patients achieving good outcomes. Keys to success are prompt diagnosis, appropriate selection of therapeutic approaches based on individual and anatomical factors, and vigilant post-treatment monitoring to prevent recurrence. As this field evolves, ongoing research and advanced imaging techniques are expected to further refine our approaches, enhancing the prognosis for transplant recipients with nephrolithiasis. The objective of this review is to delineate the risk determinants for calculi formation in patients with renal transplants and to conduct a comparative analysis of surgical interventions, specifically, PCNL and URS, in terms of their therapeutic outcomes.

## 2. Materials and Methods

We compiled a narrative review using the PubMed and EMBASE databases with no time period restriction from inception until February 2024.

The references in each included study were also reviewed, and no language restrictions were applied. We focused on surgical treatment, primarily endoscopic, and included studies regarding URS, fURS, and PCNL. Editorials, letters, conference abstracts, and studies reporting intervention with ESWL were excluded.

The data were extracted using an Excel spreadsheet: author, year, study design, sample, comparison groups, stone-free rate [SFR], puncture guide, overall complications, auxiliary procedures.

## 3. Results

### 3.1. Expanded Causes and Metabolic Abnormalities in Nephrolithiasis among Renal Transplant Recipients

#### 3.1.1. De Novo and Donor-Gifted Lithiasis

In renal transplant recipients, nephrolithiasis can originate from stones already present in the donated kidney, known as donor-gifted lithiasis, or develop due to post-transplant metabolic changes. The incidence of donor-gifted stones is rising with the increased use of living donors and improved imaging techniques, leading to a more frequent identification of preexisting stones [1,2,3,4]. Both scenarios require careful pre-transplant screening and post-transplant management to prevent complications.

Metabolic and urodynamic factors conducive to stone formation, such as urinary stasis, reflux, recurrent urinary tract infections, and renal tubular acidosis, are prevalent in transplanted kidneys [6]. These conditions, coupled with urine supersaturation and decreased inhibitor activity, significantly contribute to lithiasis [7].

#### 3.1.2. Pharmacological Influences

The immunosuppressive regimen, essential for graft survival, significantly influences the stone risk. Medications such as calcineurin inhibitors (cyclosporine, tacrolimus) and steroids impact the mineral metabolism. Cyclosporine is associated with hyperuricemia and may increase the risk of uric acid stones, whereas tacrolimus does not exhibit this side effect. Steroids, particularly in high doses during the initial post-transplant period, can lead to hypercalcemia by inducing enzymes involved in vitamin D metabolism, which increases both parathyroid hormone and fibroblast growth factor 23 levels [8,9]. This complex interaction underscores the need for careful medication management and monitoring of metabolic parameters in transplant recipients.

#### 3.1.3. Metabolic Abnormalities

Post-transplant metabolic adjustments include significant shifts in serum calcium levels. The initial drop in PTH followed by an increase due to restored vitamin D synthesis by the graft often leads to tertiary hyperparathyroidism. This condition can result in persistent hypercalcemia, affecting 10–25% of patients within months of transplantation and potentially taking years to resolve [8,9]. Other metabolic disturbances such as hyperoxaluria, hyperuricosuria, and hypocitraturia also predispose patients to stone formation. These abnormalities are often exacerbated by factors like diet, body weight, and other systemic conditions [e.g., diabetes, obesity, and metabolic syndrome] that are prevalent among transplant recipients [10,11]. The effective management of these metabolic issues is essential for preventing nephrolithiasis and preserving the renal function over the long term.

### 3.2. Diagnostic Approaches and Associated Risks of Urolithiasis in Renal Transplant Recipients

#### 3.2.1. Clinical Presentation and Localization

##### Diagnosis

The denervation of transplanted kidneys often means that pain is not a common symptom upon nephrolithiasis diagnosis in renal transplant recipients, unlike in individuals with native kidneys, where pain is usually the main indicator of nephrolithiasis. Instead, symptoms such as hematuria, obstruction, and acute kidney insufficiency tend to manifest initially in transplant patients. These symptoms can sometimes be mistakenly diagnosed as more typical complications like acute rejection or acute tubular necrosis [12].

##### Localization and Symptoms

Hematuria and acute renal failure were observed in 23.5% of the cases, while UTIs occurred in 5.9% of the patients. The most frequent locations for stones were the distal ureter [49.01%], lower calyx [17.6%], and renal pelvis [7.8%]. Notably, more than 40% of these cases were diagnosed incidentally, which likely reduced the occurrence of severe symptoms [13].

##### Images

Kidney, ureter, and bladder (KUB) ultrasound was the primary diagnostic tool used in 80.3% of these incidental cases, followed by computed tomography (CT), which accounted for 15% of the diagnoses. Magnetic resonance imaging (MRI) was used less frequently but was instrumental in complex cases where other methods provided insufficient information [13,14].

#### 3.2.2. Stone Composition

Considering the composition of kidney stones in transplant recipients, which primarily include calcium oxalate monohydrate and calcium phosphate, it is evident that these individuals share common risk factors with the general population.

These factors likely include dietary habits, inadequate hydration, urinary tract infections, hyperparathyroidism, and hypercalciuria [5].

Stone analyses indicated that calcium oxalate stones predominate, accounting for as much as 47% of the cases [4,15]. Boissier et al. analyzed 149 stones, finding that 44% were composed of oxalate monohydrate, 19% of calcium phosphate, 17% of uric acid, and 11% of struvite [16]. Similarly, Emiliani et al. observed that 23.5% of the analyzed stones consisted of calcium oxalate monohydrate, with uric acid stones present in 11.7% and struvite stones in 9.8% of the cases [13]. Additionally, uric acid stones were frequently reported, equating to the incidence rates of calcium oxalate stones in several studies [2,15,16].

#### 3.2.3. Association of Anastomosis Type and Urolithiasis Risk

The type of anastomosis showed a significant correlation with the risk of developing kidney stones. Anastomoses in neobladders and ileal conduits were associated with a high risk -18% incidence of urolithiasis-, while Lich–Gregoir anastomoses showed the lowest risk [17]. The strategic use of JJ stents and the proactive management of ureteral or infravesical obstructions were effective in preventing stone formation, suggesting that addressing urinary complications can mitigate the risk of urolithiasis.

### 3.3. Management of de Novo Graft Stones in the Recipient

#### Watchful Waiting

This strategy was adopted for asymptomatic stones under 5 mm in diameter, reflecting a cautious approach to avoid overtreatment. 

Surveillance included clinical, laboratory, and radiological assessments to monitor for potential growth or complications [1,18]. For uric acid stones, alkalinization of the urine was effective as a first-line treatment in preventing stone growth and recommended in 18% of cases involving uric acid calculi [19].

### 3.4. Endoscopic Surgical Management of de Novo Graft Stones in the Recipient

Advancements in the treatment of nephrolithiasis have leveraged technologies such as endoscopy, laser application, and minimally invasive techniques including ESWL, retrograde intrarenal surgery (RIRS), flexible ureteroscopy (fURS), PCNL. These modalities are selected based on specific stone characteristics.

#### 3.4.1. Percutaneous Nephrolithotomy

The summarized information in Table 1 covers a range of studies conducted across different countries from 2002 to 2022, including retrospective reviews, case series, and multicentric studies [1,13,19,20,21,22,23,24,25,26,27,28,29,30,31,32,33,34,35,36,37,38,39,40,41], with patient groups varying in size from single cases to 95 individuals. The stones treated with these methods ranged from small calculi of a few millimeters to large complex stones up to 50 mm.

Most studies reported high stone-free rates (SFRs), with many achieving a 100% success rate, which underscores the effectiveness of PCNL in managing renal stones. The lowest SFR was 76.90% [33], suggesting some variability influenced by procedural techniques or patient-specific factors. The guidance for puncture was predominantly ultrasound-based, though some studies used fluoroscopy or a combination of both, highlighting the importance of imaging in enhancing procedural accuracy and safety.

Complications were generally minimal, with serious adverse events being rare. Reported complications, where specified, included hematuria, urosepsis, and urinary leakage, with a few cases detailing the severity using the Clavien–Dindo classification. This indicates that while PCNL is a relatively safe procedure, careful monitoring and management of potential complications are crucial.

Auxiliary procedures such as the placement of nephrostomy tubes and double-J stents were commonly employed to ensure the safety and completeness of stone removal. The use of second-look PCNL was also noted in some cases to achieve complete stone clearance [19,21,24,33,37,40].

Overall, the gathered data indicate that PCNL, particularly when supplemented by appropriate imaging techniques, provides a highly effective treatment for renal calculi, with manageable risks.

Mini percutaneous nephrolithotomy (mPCNL) has been proposed as an effective alternative for stone removal [42]. Its smaller tract size potentially reduces damage to the renal parenchyma, which may lead to decreased bleeding risks. For instance, Ji et al. utilized laser fragmentation, a procedure with low morbidity and high efficiency [22], and Sevinc et al. opted for mPCNL rather than flexible ureteroscopy for a patient who had hydronephrosis and a large stone [38].

#### 3.4.2. Flexible Ureteroscopy

Ureteroscopic stone management is a viable approach for small stones, yielding success rates ranging from 60 to 67% [17,20]. The antegrade approach can be particularly beneficial when a nephrostomy is already in place, while retrograde techniques may be impeded by the unique anatomy of the transplant ureteric orifice and the absence of soft tissue support, which heightens the risk of ureteral perforation, especially when using a rigid scope. Hyams et al. study, involving 12 patients treated with ureteroscopy, 7 of whom via retrograde access, and 5 via antegrade access, noted the utility of specialized tools such as the Kumpe catheter and a two-wire method to facilitate a retrograde access, concluding with the placement of ureteric stents [36].

Sevinc et al. successfully treated transplanted kidney stones, with an average size of 9.2 mm, using F-URS. The procedure took, on average, 55 min, with a fluoroscopy exposure of about 58 s, leading to a complete stone clearance in all cases. The only observed side effect was hematuria, which subsided within 36 h, highlighting the treatment’s safety and effectiveness [38].

## 4. Discussion

The prevalence of nephrolithiasis in renal transplant recipients has been a subject of significant interest, with research indicating that only a third of these patients have a prior history of kidney stones, and an even smaller fraction, approximately 11%, have stones present at the time of transplantation [43]. These meta-analyses suggest a prevalence near 1% among kidney transplant recipients, though this figure includes bladder stones, which introduces ambiguity regarding the kidney origin [44].

Furthermore, the consistent presence of calcium oxalate in the analyzed stones corroborates the urine metabolic data, emphasizing a marked trend towards calcium oxalate supersaturation [4,13,43]. Emiliani et al. reported only 2% of calcium dehydrate [13]. This finding underscores the need for tailored management strategies, focusing not only on the surgical removal of stones but also on addressing underlying metabolic conditions to prevent nephrolithiasis. The cause of de novo stone formation is multifactorial and may be related to anatomical factors, infection, and/or metabolic factors. Infection stones (struvite) caused by urea-splitting organisms such as Proteus mirabilis are common in transplant patients and have been associated with encrusted ureteric stents [35].

Moreover, percutaneous nephrolithotomy, introduced in 1970, has become the standard technique for treating renal calculi larger than 2 cm or when ESWL fails [30,33]. This technique offers swift obstruction relief and infection control, proving highly effective in clearing large stone burdens in a single session [33]. However, it is noteworthy that immunosuppressive therapy in transplant patients may elevate the risk of complications like impaired wound healing and fistula formation [33]

The technique is particularly advantageous for transplanted kidneys, which are more superficially positioned, facilitating a percutaneous approach. Typically, this is done with the patient in a supine position and using an anterior calyx for access to avoid complications such as inadvertent bowel injury [30,31,39]. For treating graft lithiasis, a 16F miniature nephroscope has proven safe and effective, and a 27F rigid nephroscope is also a viable option [33]. For transplant kidneys, balloon fascial dilators are preferred for creating the access tract due to their association with reduced blood loss compared to that observed with injuries caused by mechanical sequential dilation [45]. If balloon dilation is unsuccessful, the use of Amplatz dilators or metal Alken sequential dilators may be required to navigate dense scar tissue.

Additionally, recent studies demonstrated that PCNL can be performed with minimal morbidity and without impairing the renal function in transplant recipients. For instance, Rifiaoglu et al. reported a 100% success rate using flexible scopes and baskets in anterior PCNL procedures on transplant kidneys, with the caveat of potential complications such as bleeding and delayed healing due to steroid therapy [31].

In terms of procedural outcomes, PCNL in transplanted kidneys generally appears positive, with some series reporting average operative time of 79 and 53 min, no complications, and complete stone clearance in a single session, reinforcing the efficacy of PCNL in this context when executed with precision [21,31]

On the other hand, mPCNL represents a less invasive alternative, especially relevant in cases where conditions such as anticoagulation or stone location render ESWL impracticable. With tract sizes from 8F to 16F and a ‘peel-away’ sheath, mPCNL diminishes the potential for renal damage and hemorrhage, evidenced by its success in a kidney transplant patient with a proximal ureteral stone, achieving a stone-free outcome with pneumatic or holmium–YAG laser lithotripsy [13,32,38].

Lastly, advancements in flexible ureteroscopy and its integration with laser lithotripsy have expanded its indications, especially for stones unresponsive to ESWL. In a systematic review involving 699 patients, various treatment modalities were utilized: 15.73% of the patients underwent ESWL, 26.75% were subjected to endoscopic lithotripsy or stone extraction, 18.03% were treated with PCNL, 3.14% benefited from combined approaches, and 5.01% underwent surgical lithotomy [46]. Branchereau et al. [19]. reported ureteroscopy as the most common intervention, performed in 26% of 95 patients, highlighting the heterogeneity and customization of the contemporary urological management of kidney stones.

## 5. Limitations

The narrative review format inherently relies on the authors’ interpretation and selection of the literature, which can introduce bias. This subjectivity might affect the comprehensiveness of the review and could potentially overlook conflicting or contradictory studies that might impact the overall analysis.

This review synthesized findings from diverse studies, which could vary in design, methodology, and patient populations. This could complicate the direct comparison of outcomes across the examined studies and may limit the generalizability of the review’s conclusions to all renal transplant recipients.

### 5.1. Recommendations for Cystoscopy and RIRS in Transplanted Kidney Stone Management [38]

1. Cystoscopy approach:

Start with a 30-degree lens and progress to a 70-degree lens if necessary for enhanced visualization.

Employ techniques like suprapubic pressure or bladder manipulation for difficult orifice identification.

2. Guide wire insertion:

Utilize a semirigid ureteroscope to assist in guiding wire insertion when direct angling is challenging.

Choose hydrophilic guidewires to navigate the sharp angle of the neoureteral orifice.

Difficult orifice access:

If the standard methods fail, use a 5 Fr torque catheter to help redirect the guide wire.

3. Ureteral access sheath usage:

Use a ureteral access sheath for kidney stones only after ensuring the proper guide wire placement.

4. Ureteral dilation:

Implement a DJ catheter or perform ureteral balloon dilation for ureteral obstructions to facilitate the dilation before F-URS.

In cases where the ureteral lumen is too narrow, consider inserting a second DJ catheter.

### 5.2. Recommendations for PCNL on Transplanted Kidneys [21]:

1. Positioning and access:

- Unlike PCNL in normal kidneys where access is typically through a posterior calix in the prone position, for a transplanted kidney, perform the procedure with the patient in the supine position targeting an anterior calix.

2. Preoperative imaging:

- Conduct a preoperative computed tomography (CT) scan to check for an overlying bowel and other anatomical considerations that might complicate the access. This helps in planning the safest approach for percutaneous renal access.

3. Ultrasound guidance:

- Use ultrasound guidance to assist with percutaneous access. This method helps in achieving a direct caliceal puncture and minimizes the risk of injuring overlying structures like the bowel.

- Be cautious when relying solely on fluoroscopy for percutaneous access, as it can be challenging to opacify the collecting system using a retrograde approach in transplanted kidneys.

4. Use of balloon fascial dilators:

- Consider using balloon fascial dilators which are believed to cause less blood loss by reducing the shear injury compared to sequential mechanical dilation.

- If balloon dilation is unsuccessful, Amplatz dilators or metal Alken sequential dilators might be necessary for dilating dense scar tissue surrounding the transplant site.

## 6. Conclusions

In conclusion, this review elucidates the complex risk factors associated with nephrolithiasis in renal transplant recipients and provides a comparative analysis of PCNL and URS. The findings suggest that while PCNL is highly effective for large stones, URS offers a less invasive alternative that is preferable in certain clinical scenarios. The selection of the surgical approach should be tailored to the individual patient’s needs, with a focus on minimizing morbidity and preserving the graft function. As the field of endourology advances, these insights should guide a patient-centered approach to improve outcomes for renal transplant recipients with stone disease.

## Figures and Tables

**Table 1:** Characteristics of the studies included.

Author	Year	Country	Type of Study	Procedure	Patients	Stone Size (mm)	Puncture Guidance	SFR	Auxiliary Procedure	Overall Complication Rate
Ferreira Cassini [1]	2012	Brazil	Retrospective	PCNL URS	1 3	2 to 15	None reported	Not specified	No	Not specified
Emiliani [13]	2018	Spain	Retrospective	URS PCNL	9 4	9 ± 6.5	Not specified	100% 100%	No	URS = 1 sepsis and 1 hematuria
Branchereau [19]	2018	France	Multicentre Study	URS PCNL	95	6–24 8–24	Not specified	84% 90%	3 Watchful waiting for stones < 5 mm 1 PCNL for a stone of 8 mm (technical failure of ureteroscopy)	ITU
He [21]	2007	China	Case series	mPCNL	7	5× 6 to 35 × 40	US—Middle calyceal	100%	URS for one patient	ITU
Ji [22]	2013	China	Retrospective	mPCNL	11	9–24	US—Middle calyceal	100%	Double-J stent, nephrostomy tube	None reported
Lu [23]	2002	USA	Retrospective	PCNL	3	9–25	Anterior caliceal under US and fluoroscopy	100%	Nephrostomy tube, double-J stent	None reported
He [21]	2007	China	Case series	mPCNL	7	5 × 6 to 35 × 40	US—Middle calyceal	100%	URS for one patient	ITU
Challacombe [24]	2005	UK	Case series	PCNL URS	3/21 2/21	Not specified	Not specified	100% after combined treatments	PCNL = open pyelolithotomy	NS
Palazzo [25]	2016	Italy	Case report	PCNL	2	20 and 15	US-Lower pole	100%	Double-J stent, nephrostomy	Not specified
Fonseca [26]	2021	Portugal	Case Series	PCNL	10	Not specified	Antegrade or combined access	Not specified	Not specified	Complications graded ≥II in the Clavien–Dindo classification
Kadlec [27]	2012	USA	Case Report	mPCNL	1	15		100%	10-Fr pigtail nephrostomy tube	URS = 1 Sepsis and 1 haematuria
Ketsuwan [28]	2022	Thailand	Case Report	PCNL	1	32 × 30 × 16	Used existing nephrostomy tract	100%	Not specified	minimal haematuria
Markic [29]	2016	Croatia	Case Report	mPCNL	1	None reported	Used existing nephrostomy tract	100%	Nephrostomy replaced post-op	None reporte
Oliveira [30]	2010	Portugal	Original Paper	PCNL	7	20–50	US and fluoroscopy	85.70%	No	NS
Rifaiogu [31]	2008	USA	Case Series	PCNL	15	6–40	US alone or a combination of US and fluoroscopy.	100%	Not specified	No major complications
Santillán [32]	2021	Argentina	Case Report	Combined URS + Mini-ECIRS	1	3 stones = 12.5, 13.7, 10	US	100%	JJ stent	NS
Kram-beck [33]	2008	USA	Retrospective Review	PCNL	13	2–36	US and fluoroscopy	76.90%	3 second look PCNL	1 Urosepsis (II) 1 HSV infection (II) 1 Upper GU bleeding (II)
Sarier [34]	2018	Turkey	Retrospective Review	PCNL URS	1 19	4–29 4–25	Not specified	91%	2 second-look procedures	4.5% (one case of UTI post-flexible URS)
Stravodimos [35]	2012	Greece	Retrospective Analysis	PCNL	3	4–25	US and fluoroscopy	100%	Nephrostomy, Double-J stent	NS
Hyams [36]	2012	Multicenter	Multicenter Study	PCNL URS	5 7	4–15	Fluoroscopy	100%	Double-J stents, nephrostomy	Minor complications (fistula, stent encrustation)
Sevinc [38]	2015	Turkey	Retrospective Analysis	URS mPCNL	5 1	7.5–11 22	Not specified	100%	DJ stent placement	Transient hematuria
Mamarelis [37]	2014	Greece	Retrospective	PCNL	9	4–25	Not specified	100%	2 ESWL	NS

## Data Availability

No new data were created or analyzed in this study. Data sharing is not applicable to this article.
